# Gender, psychological distress, and subjective well-being two years after the onset of the COVID-19 pandemic in Spain

**DOI:** 10.1590/0102-311XEN141523

**Published:** 2024-03-22

**Authors:** M. Pilar Matud, Amelia Díaz, Mª. José del Pino, Demelza Fortes, Ignacio Ibáñez

**Affiliations:** 1 Universidad de La Laguna, San Cristóbal de La Laguna, España.; 2 Universidad de Valencia, Valencia, España.; 3 Universidad Pablo de Olavide, Sevilla, España.

**Keywords:** COVID-19, Psychological Distress, Subjective Well-Being, Gender, COVID-19, Distrés Psicológico, Bienestar Subjetivo, Género, COVID-19, Angústia Psicológica, Bem-estar Subjetivo, Gênero

## Abstract

This study aimed to examine gender differences in distress and well-being two years after the onset of the COVID-19 pandemic, analyzing risk and protective factors for psychological distress and subjective well-being. It is a repeated cross-sectional study with a sample of 1,588 women (50%) and men (50%) from the general Spanish population aged 18-74 years who were assessed online by seven questionnaires and scales. Descriptive, variance, and hierarchical multiple regression analyses were performed. From February to April 2022, 57.4% of women and 38.7% of men had psychological distress, percentages that totaled 50.5% and 41.5%, respectively, from October 2022 to February 2023. Women also had greater perceived vulnerability to diseases, more negative feelings, and lower affect balance, resilience, and self-esteem than men. The most important predictors of greater psychological distress refer to lower self-esteem, resilience, and social support and higher perceived vulnerability to diseases. Other statistically significant predictors included lower educational level in women and neither being married nor living with a partner in men. Lower self-esteem also best predicted lower subjective well-being, with lower social support and lower resilience also constituting significant predictors. Moreover, lower educational level and higher perceived vulnerability to diseases statistically and significantly predicted lower subjective well-being in women, as did not being a student in men. We conclude that psychological distress remains greatly prevalent in Spain two years after the beginning of the COVID-19 pandemic, especially in women.

## Resumen

Este estudio tuvo como objetivo analizar las diferencias de género en distrés psicológico y en el bienestar a dos años del inicio de la pandemia de COVID-19, analizando factores de riesgo y de protección para distrés psicológico y bienestar subjetivo. Se trata de un estudio transversal repetido con una muestra de 1.588 individuos de la población general de España, 50% mujeres y 50% hombres, con edades comprendidas entre 18 y 74 años. Los participantes fueron evaluados en línea mediante 7 cuestionarios y escalas. Se realizaron análisis descriptivos, de varianza y de regresión múltiple jerárquica. Entre febrero y abril del 2022, el 57,4% de las mujeres y el 38,7% de los hombres presentaron distrés psicológico, con porcentajes del 50,5% y 41,5%, respectivamente, entre octubre del 2022 y febrero del 2023. Las mujeres también presentaron una mayor vulnerabilidad percibida a la enfermedad, más sentimientos negativos, menos equilibrio afectivo y menor resiliencia y autoestima que los hombres. Los indicadores más importantes de un mayor distrés psicológico fueron la baja autoestima, la baja resiliencia, el escaso apoyo social y una mayor vulnerabilidad percibida a la enfermedad. Otros indicadores estadísticamente significativos fueron los siguientes: bajo nivel de educación entre las mujeres y no estar casado o no vivir con una compañera entre los hombres. La baja autoestima también fue el mejor indicador de un bajo bienestar subjetivo; además, el escaso apoyo social y la baja resiliencia también fueron indicadores importantes. Además, el bajo nivel de educación y la alta vulnerabilidad percibida a la enfermedad fueron indicadores estadísticamente significativos de bajo bienestar subjetivo entre las mujeres y de no ser estudiantes entre los hombres. Concluimos que el distrés psicológico sigue siendo muy prevalente en España dos años después del inicio de la pandemia de COVID-19, especialmente entre las mujeres.

## Resumo

Este estudo teve como objetivo analisar as diferenças de gênero em estresse psicológico e no bem-estar dois anos após o início da pandemia da COVID-19, analisando fatores de risco e de proteção para estresse psicológico e bem-estar subjetivo. Este foi um estudo transversal repetido com amostra de 1.588 indivíduos da população geral da Espanha - 50% do sexo feminino e 50% do sexo masculino, com idade entre 18 e 74 anos. Os participantes foram avaliados *online* por meio de sete questionários e escalas. Foram realizadas análises descritivas, de variância e de regressão múltipla hierárquica. Entre fevereiro e abril de 2022, 57,4% das mulheres e 38,7% dos homens apresentaram estresse psicológico, com porcentagens de 50,5% e 41,5%, respetivamente, entre outubro de 2022 e fevereiro de 2023. As mulheres também apresentaram maior vulnerabilidade percebida à doença, mais sentimentos negativos, menor equilíbrio afetivo e menor resiliência e autoestima do que os homens. Os indicadores mais importantes de maior estresse psicológico foram baixa autoestima, baixa resiliência, baixo apoio social e maior vulnerabilidade percebida à doença. Outros indicadores estatisticamente significativos foram: baixo nível de escolaridade entre as mulheres e não ser casado ou não viver com uma companheira entre os homens. Baixa autoestima também foi o melhor indicador de baixo bem-estar subjetivo; além disso, baixo apoio social e baixa resiliência também foram indicadores significativos. Além disso, baixo nível de escolaridade e alta vulnerabilidade percebida à doença foram indicadores estatisticamente significativos de baixo bem-estar subjetivo entre as mulheres e de não ser estudante entre os homens. Concluímos que estresse psicológico ainda é muito prevalente na Espanha dois anos após o início da pandemia da COVID-19, principalmente entre as mulheres.

## Introduction

The COVID-19 pandemic has generated a major public health problem worldwide, having especially impacted South America ^1^ ^,^ ^2^ and, during 2020, Spain, one of the most affected European countries ^3^. In addition to the health threat due to the new coronavirus, people had to face other circumstances related to the pandemic, such as periods of confinement, isolation, and social distancing, as well as the state of economic uncertainty due to the pandemic and the measures to contain it ^4^ ^,^ ^5^ ^,^ ^6^, which generated, among other problems, the temporary closure of educational centers, loss of employment, and saturation of health services. This has increased mental health problems and needs as a direct and indirect effect of the COVID-19 pandemic ^7^. Global and national responses to COVID-19 have shown unique and rapidly changing challenges to health promotion and protection, and COVID-19 has increased existing social and gender inequalities and impacted women differently than men ^8^.

Although research has shown an increase in mental health problems in the general population during the beginning of the pandemic ^6^ ^,^ ^7^ ^,^ ^9^ ^,^ ^10^ ^,^ ^11^ ^,^ ^12^, the evolution of mental health throughout the pandemic remains unclear. Studies ^12^ ^,^ ^13^ and mental health responses have shown heterogeneity, including different trajectories of psychological distress and symptoms ^10^ ^,^ ^14^. While some longitudinal studies have shown an increase in the prevalence of psychological distress immediately following the onset of the COVID-19 pandemic - followed by significant decreases a few months later and reaching pre-pandemic levels after the relaxation of restrictions by mid-2020 ^9^, increasing again during the second pandemic wave ^15^ -, other studies found that deteriorating mental health persisted across the first year of the pandemic ^13^, although deterioration varied according to sociodemographic factors ^11^ ^,^ ^13^. Studies have shown consistent evidence that the impact has been greater in women ^6^ ^,^ ^7^ ^,^ ^9^ ^,^ ^10^ ^,^ ^11^ ^,^ ^13^ ^,^ ^15^ ^,^ ^16^ ^,^ ^17^ ^,^ ^18^, young people ^6^ ^,^ ^7^ ^,^ ^9^ ^,^ ^10^ ^,^ ^11^ ^,^ ^18^, students ^6^ ^,^ ^17^ ^,^ ^18^, and unemployed persons ^6^ ^,^ ^17^ ^,^ ^19^. Other risk factors for poorer mental health include higher perceived threat ^19^ ^,^ ^20^ ^,^ ^21^ and lower resilience ^10^ ^,^ ^15^, self-esteem ^16^ ^,^ ^17^ ^,^ ^20^ ^,^ ^22^, and social support ^16^ ^,^ ^17^ ^,^ ^23^.

Despite the many studies on the effect of COVID-19 on mental health, most were carried out within the first year of the pandemic, and very little is known on how psychological distress and subjective well-being have evolved years after the onset of the COVID-19 pandemic. Therefore, the objectives of this study include (i) determining the existence of differences between women and men in psychological distress; cognitive and affective components of subjective well-being; and perceived vulnerability to diseases, resilience, self-esteem, and social support two years after the onset of the COVID-19 pandemic and at the end of the first wave of the pandemic and (ii) assessing the relevance of sociodemographic characteristics and perceived vulnerability to diseases, resilience, self-esteem, and social support on psychological distress and subjective well-being in women and men two years after the onset of the COVID-19 pandemic and at the end of the first wave of the pandemic. Based on previous literature, the following hypotheses are proposed:

1) Women will endure greater psychological distress than men.

2) High self-esteem, resilience, and social support and low perceived vulnerability to diseases will act as protective factors against psychological distress in women and men.

## Methods

### Study design and sample

This repeated cross-sectional study was carried out with a nonprobabilistic sample of 1,588 people (50% women) from the general Spanish population aged from 18 to 74 years. Almost one third of participants (230 women and 230 men) were tested two years after the beginning of the COVID-19 pandemic (from February to April 2022), and the remaining (564 women and 564 men) were tested two years after the end of the first wave of COVID-19 in Spain from October 2022 to February 2023. The second data collection was independent of the first. The number of women and men in each data collection stage was controlled to total the same amount and that participants would avoid significantly differing in their sociodemographic characteristics of age, educational level, marital status, number of children, and occupation (Table 1). The data were obtained by an online questionnaire using a Google form in which, after information on sociodemographic characteristics was collected, the questionnaires and scales described in the *Study Variables* subsection were included. The sampling was performed by convenience and the snowball technique, with the collaboration of students from three Spanish universities (who received course credits for their participation in data collection) and the members of the research group.

The main sociodemographic characteristics and the comparisons between women and men in each test pass are shown in Table 1, in which the similarity of women and men according to these variables can be seen. Participants’ mean age totaled just over 33 years, and more than half of the sample (70.2%) had no children, with a range from 0 to 5. Despite the diversity in their sociodemographic characteristics, participants more often had secondary education or vocational training, with fewer people having only basic education. Almost half of the sample (48.9%) were single and did not live with a partner, and a similar percentage (46.6%) was married or living with a partner, with a minority being separated, divorced, or widowed. Most often, participants were employed (half of the sample), and practically a third were students.

**Table 1:** Sociodemographic characteristics of the women and men groups according to study times.

Characteristics	Time 1					Time 2				
	Men (n = 230)		Women (n = 230)		χ^2^	Men (n = 564)		Women (n = 564)		χ^2^
	n	%	n	%		n	%	n	%	
Educational level					2.61					1.07
Elementary studies	18	7.8	10	4.3		37	6.6	41	7.3	
Compulsory secondary studies	20	8.7	20	8.7		57	10.1	48	8.5	
High school/Professional training	94	40.9	102	44.3		284	50.4	283	50.2	
University degree	98	42.6	98	42.6		186	33.0	192	34.0	
Marital status					0.40					0.00
Never married	110	47.8	106	46.1		280	49.6	280	49.6	
Married/Living with a partner	107	46.5	113	49.1		260	46.1	260	46.1	
Separated/Divorced/Widowed	13	5.7	11	4.8		24	4.3	24	4.3	
Occupation					0.21					1.27
Working	113	49.1	114	49.6		291	51.6	291	51.6	
Unemployed	19	8.3	18	7.8		41	7.3	43	7.6	
Retired	14	6.1	12	5.2		28	5.0	29	5.1	
Student	83	36.1	85	37.0		191	33.9	193	34.2	
Other	1	0.4	1	0.4		13	2.3	8	1.4	
	M	SD	M	SD	*t*	M	SD	M	SD	*t*
Age	33.50	14.74	33.67	14.70	-0.12	33.25	15.30	33.04	15.09	0.24
Number of children	0.47	0.87	0.50	0.89	-0.32	0.57	0.98	0.60	0.98	-0.40

M: mean; SD: standard deviation.

Note: Time 1 (February to April 2022); Time 2 (October 2022 to February 2023).

### Study variables

In addition to sociodemographic characteristics, seven questionnaires and scales were used as described as follows. The following dependent variables were used: psychological distress - which was assessed using the Spanish version of the *Goldberg General Health Questionnaire* (GHQ-12) ^24^ - and subjective well-being - which was measured by two instruments: the *Positive and Negative Experience Scale* (SPANE) ^25^, which assesses the affective component of subjective well-being, and the *Satisfaction With Life Scale* (SWLS) ^26^, which measures the cognitive component.

GHQ-12 is a brief screening instrument consisting of 12 questions with four response alternatives that has been widely used to assess psychological distress ^9^ ^,^ ^11^ ^,^ ^21^. In this study, its items were assessed (i) according to the Likert method, which assigns a score of 0-1-2-3 and offers a mean symptom score indicating a central average for the population ^11^, and (ii) according to the GHQ method, which assigns weights of 0-0-1-1, in which a score was used to categorically analyze psychological distress using a threshold ≥ 4, as it has been considered the best for discriminating cases from noncases (sensitivity = 81.7 and specificity = 85.4) ^27^ and has been used in other studies to determine the proportion of people with clinically significant psychological distress during the COVID-19 pandemic ^9^ ^,^ ^11^ ^,^ ^15^. For the sample in this study, the 12 items of the questionnaire had a high internal consistency, i.e., 0.90 for the Likert-type score and 0.89 for the GHQ score.

The Spanish version of the SPANE was used to assess positive and negative affect and affect balance. SPANE is a scale consisting of 12 items, six assessing positive experiences (SPANE-P) and six, negative experiences (SPANE-N). The positive and negative scales are scored separately, and the two scores are combined to measure the affect balance (SPANE-B) score, which is obtained by subtracting the SPANE-N score from the SPANE-P score. For the sample in this study, Cronbach’s α for positive feelings totaled 0.91 and for negative feelings, 0.84.

The cognitive component of subjective well-being was assessed using the Spanish version of the SWLS, which is designed to assess persons’ overall judgment of their satisfaction with life. The scale contains five items with a 7-point Likert-type response format ranging from 1 (strongly disagree) to 7 (strongly agree), in which higher scores indicate higher life satisfaction. For the sample in this study, Cronbach’s α coefficient for the five items totaled 0.87.

Perceived vulnerability to disease was assessed using the Spanish version ^28^ of the *Perceived Vulnerability to Disease Questionnaire* (PVDQ) ^29^. The questionnaire consists of 15 items that measure beliefs associated with personal susceptibility to infectious diseases and perceptions and behaviors in situations with the potential risk of pathogen transmission. The response scale is a 7-point Likert-type scale (from “strongly disagree” to “strongly agree”). For the sample in this study, Cronbach’s α coefficient for the 15 items totaled 0.77.

Resilience was assessed using the Spanish version of the *Brief Resilience Scale* (BRS) ^30^, an instrument consisting of six items that assess the person’s ability to recover from stress. The response scale consists of a 5-point instrument ranging from 1 (strongly disagree) to 5 (strongly agree). For the sample in this study, Cronbach’s α coefficient totaled 0.80.

The Spanish version of the *Rosenberg Self-Esteem Scale* (RSES) ^31^ was used to assess self-esteem. This 10-item scale evaluates global self-esteem. The response format has four point ranging from 0 (strongly agree) to 3 (strongly disagree), with higher scores indicating higher levels of self-esteem. For the sample in this study, Cronbach’s α totaled 0.87.

Social support was assessed using the *Social Support Scale* (SSS) ^32^. This scale consists of 12 items that measure perceived social support in various areas, such as work, family, esteem, and companionship. The response format is a 4-point Likert-type scale ranging from 0 (never) to 3 (always), with higher scores indicating higher levels of social support. For the sample in this study, Cronbach’s α coefficient totaled 0.93.

### Statistical analysis

Descriptive analyses were computed to describe sociodemographic characteristics and the distribution of study variables, and their internal consistency was computed using Cronbach’s α. Comparisons between women and men were computed using Pearson’s chi-squared tests for categorical variables and the Student’s t-test for quantitative ones. To determine the existence of differences between women and men and in the two collection times, ten 2x2 between-subjects analyses of variance (ANOVA) were performed. Gender (women and men) and time (Time 1: February to April 2022 and Time 2: October 2022 to February 2023) were used as independent variables and study variables, as dependent variables. Hierarchical multiple regression analyses were conducted to determine the relevance of sociodemographic characteristics, perceived vulnerability to disease, resilience, self-esteem, and social support on women’s and men’s psychological distress and subjective well-being. Psychological distress scores were considered as the criterion in the first regression analyses and subjective well-being scores, in the second regression analyses. The subjective well-being score was obtained by summing the scores on the affective (affect balance) and cognitive components (life satisfaction) of subjective well-being. In the first step (Model 1) of each regression analysis, age and number of children were included as quantitative variables; educational level, as an ordinal variable with five levels (no studies, elementary studies, compulsory secondary studies, high school/professional, and university degree); and marital status and profession, as dummy variables. Marital status was coded as 0 for never married, separated/divorced, and/or widowed persons, and 1 for those married or living with a partner. Overall, two dummy variables were used for occupation: (i) unemployment, coded as 1 for those who were unemployed and as 0 for the remaining occupational categories (students, retired, working, others); (ii) student, which was coded as 1, and as 0 for the remaining the occupational categories (unemployed, retired, working, others). In step 2 (Model 2), the score on perceived vulnerability to disease was added, and in the third step (Model 3), scores on resilience, self-esteem, and social support were included.

Statistical analyses were performed on IBM SPSS, version 22.0 (https://www.ibm.com/), and, except for comparisons, were performed independently for women and men, with all the data disaggregated by gender.

### Ethical considerations

This research complied with the ethical criteria of the *Declaration of Helsinki* and the American Psychological Association ethical principles. All participants gave their informed consent before completing the questionnaires and scales and could withdraw their participation in this study at any time. This research was positively evaluated by the Research Ethics and Animal Welfare Committee of the University of La Laguna, Spain (study approval n. CEIBA2022-3136). This research received no funding.

## Results

At Time 1 (February to April 2022), more than half of the women (57.4%) had scores that exceeded the threshold for distressed cases, whereas the percentage for men totaled 38.7%, showing statistically significant differences, χ^2^ (1, N = 460) = 16.10, p < 0.001. Time 2 (from October 2022 to February 2023), classified half of the women, 50.5%, and 41.5% of the men as psychologically distressed, also configuring statistically significant differences, χ^2^ (1, N = 1,128) = 9.28, p = 0.002. Differences in percentages between the two study times were statistically insignificant for men, χ^2^ (1, N = 794) = 0.53, p = 0.467, whereas they were marginally statistically significant for women, χ^2^ (1, N = 794) = 3.08, p = 0.079.

Table 2 shows the results of 2-factor ANOVAs for participants’ gender (women, men) and time as between-subjects factors, using questionnaire and results as its dependent variable. The ANOVA test showed neither statistically significant differences for the gender x time interaction nor for main effect of time, except if the analysis considered social support as its dependent variable, in which the main effect of time also obtained statistically significant differences, in which the estimated marginal means totaled 25.95 (95% confidence interval [95% CI]: 25.18-26.71) at Time 1 and 25.03 (95%CI: 24.54-25.52) at Time 2. The estimated marginal means of the main effect of gender totaled 24.99 (95%CI: 24.35-25.63) in men and 25.99 (95%CI: 25.35-26.63) in women.

**Table 2:** Means (M), standard deviations (SD), and 2-way analysis of variance statistics for study variables.

Variables	Men		Women		ANOVA			
	M	SD	M	SD	Effect	F ratio	df	η^2^
Psychological distress								
Time 1	13.46	6.76	16.35	7.46	Gender	40.19 *	1.1584	0.025
Time 2	13.39	6.14	15.25	7.06	Time	2.46	1.1584	0.002
Gender x Time					Gender x Time	1.85	1.1584	0.001
Perceived vulnerability to diseases								
Time 1	46.86	13.12	52.73	14.40	Gender	50.98 *	1.1584	0.031
Time 2	47.67	12.71	52.47	14.03	Time	0.14	1.1584	0.000
Gender x Time					Gender x Time	0.51	1.1584	0.000
Negative feelings								
Time 1	15.88	4.15	17.32	4.47	Gender	35.10 *	1.1584	0.022
Time 2	15.62	4.59	17.14	4.58	Time	0.80	1.1584	0.001
Gender x Time					Gender x Time	0.02	1.1584	0.000
Positive feelings								
Time 1	21.15	4.46	21.19	4.23	Gender	0.06	1.1584	0.000
Time 2	21.34	4.40	21.43	4.20	Time	0.80	1.1584	0.001
Gender x Time					Gender x Time	0.01	1.1584	0.000
Affect balance								
Time 1	5.27	7.66	3.87	7.61	Gender	10.86 **	1.1584	0.007
Time 2	5.72	7.93	4.29	7.73	Time	1.03	1.1584	0.001
Gender x Time					Gender x Time	0.00	1.1584	0.000
Life satisfaction								
Time 1	22.99	6.36	22.05	6.65	Gender	3.39	1.1584	0.002
Time 2	23.09	6.59	22.67	6.80	Time	0.95	1.1584	0.001
Gender x Time					Gender x Time	0.49	1.1584	0.000
Subjective well-being								
Time 1	28.26	12.53	25.92	12.72	Gender	8.72 **	1.1584	0.005
Time 2	28.81	12.77	26.96	13.03	Time	1.26	1.1584	0.001
Gender x Time					Gender x Time	0.12	1.1584	0.000
Resilience								
Time 1	16.10	4.35	14.26	4.82	Gender	70.12 *	1.1584	0.042
Time 2	16.52	4.26	14.24	4.50	Time	0.62	1.1584	0.000
Gender x Time					Gender x Time	0.82	1.1584	0.001
Self-esteem								
Time 1	20.19	5.23	19.48	5.59	Gender	5.53 ***	1.1584	0.003
Time 2	20.19	5.20	19.49	5.63	Time	0.01	1.1584	0.000
Gender x Time					Gender x Time	0.00	1.1584	0.000
Social support								
Time 1	25.75	7.65	26.14	8.63	Gender	4.72 ***	1.1584	0.003
Time 2	24.22	8.39	25.84	8.48	Time	3.93 ***	1.1584	0.002
Gender x Time					Gender x Time	1.78	1.1584	0.001

df: degree of freedom.

Note: Time 1 (February to April 2022); Time 2 (October 2022 to February 2023).

* p < 0.001;

** p < 0.01;

*** p < 0.05.

All analyses obtained statistically significant main effects for gender, except if they considered positive feelings and life satisfaction as their dependent variables, which resulted in no statistically significant effect. Women showed higher mean scores than men for psychological distress as men’s score totaled 13.43 (95%CI: 12.91-13.95) and women, 15.80 (95%CI: 15.28-16.32); in perceived vulnerability to disease, with the score in men being 47.27 (95%CI: 46.23-48.30) and in women 52.60 (95%CI: 51.57-53.64); in negative feelings, where the score in men was 15.75 (95%CI: 15.41-16.10) and in women 17.23 (95%CI: 16.88-17.57). Men showed higher mean scores than women in affect balance as men scored 5.50 (95%CI: 4.90-6.09) and women, 4.08 (95%CI: 3.48-4.68); in subjective well-being as men scored 28.53 (95%CI: 27.55-29.52) and women, 26.44 (95%CI: 25.46-27.42); in resilience as men scored 16.31 (95%CI: 15.97-16.45) and women, 14.25 (95%CI: 13.91-14.59); and in self-esteem as men scored 20.19 (95%CI: 19.77-20.61) and women, 19.49 (95%CI: 19.07-19.90).

Table 3 shows the main results of the hierarchical multiple linear regression analyses predicting psychological distress scores for women and men. Model 1, which only included sociodemographic variables, showed a statistically significant R^2^, and women with greater psychological distress were younger and less educated, whereas men with greater psychological distress were neither married, lived with their partner, nor were employed. Adding the perceived vulnerability to disease score to the regression equation (Model 2) resulted in a statistically significant R^2^ change in both groups, with greater psychological distress in women and men with greater perceived vulnerability to diseases. The addition of resilience, self-esteem, and social support scores (Model 3) resulted in a statistically significant change in R^2^ (R^2^ change = 0.39 in women and R^2^ change = 0.35 in men). In the final model, with all variables in the equation regression, the most important variable predicting women’s and men’s psychological distress refer to lower self-esteem, followed by lower resilience, less social support, and higher perceived vulnerability to disease. In women, another statistically significant predictor refers to lower educational level, whereas, in men, to neither being married nor living with a partner. The final model accounted for 47.2% of the variance in psychological distress in women and 46% in men.

**Table 3:** Hierarchical regression results for psychological distress for women and men.

Variables	Women			Men		
	β	R^2^	ΔR^2^	β	R^2^	ΔR^2^
Model 1		0.05	0.05 *		0.05	0.05 *
Age	-0.23 *			-0.10		
Number of children	0.02			0.00		
Educational level	-0.12 **			-0.02		
Married/Living with a partner	0.02			-0.11 **		
Unemployed	0.04			0.07 ***		
Student	-0.05			0.06		
Model 2		0.09	0.04 *		0.11	0.06 *
Age	-0.22 *			-0.13 ***		
Number of children	0.01			0.00		
Educational level	-0.13 *			-0.03		
Married/Living with a partner	0.00			-0.11 **		
Unemployed	0.04			0.07		
Student	-0.03			0.05		
Perceived vulnerability to diseases	0.21 *			0.25 *		
Model 3		0.48	0.39 *		0.46	0.35 *
Age	-0.07			-0.09		
Number of children	0.02			0.06		
Educational level	-0.06 ***			0.03		
Married/Living with a partner	0.06			-0.07 ***		
Unemployed	0.01			0.03		
Student	-0.05			0.02		
Perceived vulnerability to diseases	0.08 **			0.10 *		
Resilience	-0.19 *			-0.13 *		
Self-esteem	-0.50 *			-0.50 *		
Social support	-0.13 *			-0.10 **		

β: standardized coefficient; ΔR^2^: R^2^ change.

* p < 0.001;

** p < 0.01;

*** p < 0.05.

Table 4 shows the main results of the hierarchical multiple linear analyses predicting subjective well-being for women and men. As can be seen, Model 1 obtained a statistically significant R^2^, in which older women, those with a higher educational level or who were married or lived with a partner had higher subjective well-being. Married men or those living with a partner also had higher subjective well-being, as did those who were employed. The addition of perceived vulnerability to disease scores in Model 2 statistically and significantly changed R^2^ in both groups, with higher subjective well-being in women and men with lower perceived vulnerability to diseases. The addition of resilience, self-esteem, and social support scores (Model 3) importantly, statistically, and significantly increased R^2^ (0.50 in women and 0.52 in men). Beta values in the final model with all variables in the regression equation indicated that self-esteem constituted the variable most associated with women’s and men’s subjective well-being. In the women’s group, the second most relevant predictor of higher subjective well-being referred to greater social support, followed by higher resilience, higher educational level, and lower perception of vulnerability to diseases. In men, the second most relevant predictor of greater subjective well-being referred to higher resilience, higher social support, and being a student. In the final model, the adjusted R^2^ value of 0.590 in women and 0.586 in men indicated explained more than half of the variance in subjective well-being.

**Table 4:** Hierarchical regression results for subjective well-being for women and men.

Variables	β	R^2^	ΔR^2^	β	R^2^	ΔR^2^
Model 1		0.06	0.06 *		0.04	0.04 *
Age	0.20 *			0.05		
Number of children	-0.02			0.07		
Educational level	0.14 *			0.00		
Married/Living with a partner	0.10 **			0.10 ***		
Unemployed	-0.07			-0.08 ***		
Student	0.07			0.02		
Model 2		0.10	0.04 *		0.07	0.03 *
Age	0.18 **			0.08		
Number of children	-0.01			0.07		
Educational level	0.14 **			0.01		
Married/Living with a partner	0.11 **			0.10 ***		
Unemployed	-0.07 ***			-0.07 ***		
Student	0.05			0.03		
Perceived vulnerability to diseases	-0.20 *			-0.19 *		
Model 3		0.60	0.50 *		0.59	0.52 *
Age	0.03			0.04		
Number of children	0.01			0.01		
Educational level	0.06 ***			-0.04		
Married/Living with a partner	0.04			0.03		
Unemployed	-0.02			-0.03		
Student	0.06			0.07 ***		
Perceived vulnerability to diseases	-0.06 ***			0.00		
Resilience	0.17 *			0.21 *		
Self-esteem	0.56 *			0.53 *		
Social support	0.21 *			0.20 *		

β: standardized coefficient; ΔR^2^: R^2^ change.

* p < 0.001;

** p < 0.01;

*** p < 0.05.

## Discussion

The COVID-19 pandemic has been considered one of the most impactful mass disability events in recent public health history ^33^, greatly impacting the health and well-being of the general population, albeit with important individual and group differences, including gender. Although the direct effects of the COVID-19 pandemic seem to have more greatly affected men with higher mortality rates ^34^ ^,^ ^35^, its indirect effects have affected women more ^35^ ^,^ ^36^, with greater deterioration in their mental health after the onset of the COVID-19 pandemic than in men ^37^ ^,^ ^38^ as the pandemic intensified preexisting inequalities between women and men ^7^ ^,^ ^8^ ^,^ ^35^ ^,^ ^36^. This research aims to contribute to the literature by providing evidence of gender differences in psychological distress and subjective well-being two years after the onset of the COVID-19 pandemic. Results show that, in Spain, more than two years after the beginning of the pandemic, half of the assessed women showed psychological distress (a percentage that totaled 41.5% in men), thus confirming the first hypothesis of this study. These percentages exceed those in a national longitudinal study carried out in the United Kingdom ^15^ that used the same assessment instrument and threshold and found 20.7% of its evaluated outcome before the COVID-19 pandemic, 29.8% during the first wave of the pandemic, 21.5% in September 2020, and 27.6% in January 2021 during the second pandemic wave ^9^ ^,^ ^15^. However, the percentages in this study are lower than those found in Spain during the second wave of the pandemic, which totaled 49.5% in men and 63.4% in women. Despite important differences in occupation, the percentage of women with psychological distress exceeded that of men in all groups ^17^. Additionally, in studies carried out in Spain during the first wave, women’s psychological distress exceeded that of men. Moreover, the differences between women and men increased after the state of alarm during lockdown, and psychological distress increased in women as the first wave of the pandemic progressed (but not in men) ^16^. This study also found that women had more negative feelings, less affect balance, greater perception of vulnerability to the disease, less resilience and lower self-esteem than men, with no differences between women and men in positive feelings or life satisfaction, which constitutes the cognitive component of subjective well-being. Furthermore, women had greater perceived social support than men, although social support scores decreased at Time 2 in both genders (more than two years after the end of the first wave of the pandemic) in relation to Time 1 (two years after the beginning of the pandemic).

This analysis of risk and protective factors for psychological distress and subjective well-being showed that the greatest differences between women and men referred to sociodemographic factors. However, including self-esteem, resilience, and social support in the regression equation decreased the relevance of these factors, except for educational level in women as higher educational level configured a protective factor against psychological distress and subjective well-being. Marital status played a similar role in men, with less psychological distress in married men or those living with a partner and higher subjective well-being in students. High self-esteem configured the most relevant mental health protective factor in women and men, results that agree with those of other studies ^16^ ^,^ ^17^ ^,^ ^20^ ^,^ ^22^. Higher resilience and social support also constituted important protective factors for mental health, as in other studies ^10^ ^,^ ^15^ ^,^ ^16^ ^,^ ^17^ ^,^ ^23^ ^,^ ^39^. Perceived vulnerability to diseases constituted a risk factor for psychological distress in women and men and for women’s lower subjective well-being. Therefore, these results confirm the second hypothesis of this study, although only partially since perceived vulnerability to diseases only configured a risk factor for psychological distress in women.

The interpretation of the results of this research should consider the limitations of this study due to its non-probabilistic sample, its cross-sectional design, the fact that all measurements stem from self-reports, and all persons making up the sample resided in Spain. Another limitation includes this study only analyzing gender despite the evidence that COVID-19 also increased other social inequalities, including race and poverty ^40^. Racism, worse social and economic conditions, and the effects of COVID-19 have increased hospitalization, morbidity, and mortality rates among black people ^40^ ^,^ ^41^ ^,^ ^42^ ^,^ ^43^, with their mental health having worsened more than that of white people ^44^, widening disparities in physical and mental health between racial/ethnic groups ^42^ ^,^ ^44^.

## Public health implications

This study provides important information on psychological distress and subjective well-being in the Spanish adult population two years after the onset of the COVID-19 pandemic. Knowledge of at-risk groups and mental health risk and protective factors can guide surveillance efforts and target interventions to improve the population’s mental health and well-being.
